# Treatment Outcome of Patients with Buruli Ulcer Disease in Togo

**DOI:** 10.1371/journal.pntd.0004170

**Published:** 2015-10-16

**Authors:** Marcus Beissner, Nathalie Arens, Franz Wiedemann, Ebekalisaï Piten, Basile Kobara, Malkin Bauer, Karl-Heinz Herbinger, Kossi Badziklou, Abiba Banla Kere, Thomas Löscher, Jörg Nitschke, Gisela Bretzel

**Affiliations:** 1 Department of Infectious Diseases and Tropical Medicine (DITM), University Hospital, Ludwig-Maximilians-University, Munich, Germany; 2 German Leprosy and Tuberculosis Relief Association, Togo Office (DAHW-T), Lomé, Togo; 3 Centre Hospitalier Régional Maritime, Tsévié (CHR-Tsévié), Togo; 4 Programme National de lutte contre l'Ulcère de Buruli, la Lèpre et le Pian (PNLUB-LP), Lomé, Togo; 5 Institut National d’Hygiène (INH), Ministère de la Santé, Lomé, Togo; Fondation Raoul Follereau, FRANCE

## Abstract

**Background:**

Following introduction of antimycobacterial treatment of Buruli ulcer disease (BUD), several clinical studies evaluated treatment outcomes of BUD patients, in particular healing times, secondary lesions and functional limitations. Whereas recurrences were rarely observed, paradoxical reactions and functional limitations frequently occurred. Although systematic BUD control in Togo was established as early as 2007, treatment outcome has not been reviewed to date. Therefore, a pilot project on post-treatment follow-up of BUD patients in Togo aimed to evaluate treatment outcomes and to provide recommendations for optimization of treatment success.

**Methodology/Principal Findings:**

Out of 199 laboratory confirmed BUD patients, 129 could be enrolled in the study. The lesions of 109 patients (84.5%) were completely healed without any complications, 5 patients (3.9%) had secondary lesions and 15 patients (11.6%) had functional limitations. Edema, category III ulcers >15cm, healing times >180 days and a limitation of movement at time of discharge constituted the main risk factors significantly associated with BUD related functional limitations (*P*<0.01). Review of all BUD related documentation revealed major shortcomings, in particular concerning medical records on adjuvant surgical and physiotherapeutic treatment.

**Conclusions/Significance:**

This study presents the first systematic analysis of treatment outcome of BUD patients from Togo. Median times to healing and the absence of recurrences were in line with findings reported by other investigators. The percentage of functional limitations of 11.6% was lower than in other studies, and edema, category III ulcers, healing time >180 days and limitation of movement at discharge constituted the main risk factors for functional limitations in Togolese BUD patients. Standardized treatment plans, patient assessment and follow-up, as well as improved management of medical records are recommended to allow for intensified monitoring of disease progression and healing process, to facilitate implementation of therapeutic measures and to optimize treatment success.

## Author Summary

Buruli ulcer disease (BUD) is a mycobacterial skin disease which leads to large ulcerations and causes disabilities in approximately 25% of the patients. Treatment consists of antimycobacterial drugs, complemented by surgery and physiotherapy if necessary. Available data on treatment outcome of BUD patients suggest that recurrences are rare; paradoxical reactions and functional limitations, however, frequently occur. BUD control in Togo was introduced already in 2007, but treatment outcome has not yet been reviewed. Therefore, a clinical follow-up study assessed a cohort of 129 BUD patients at least six months after the end of treatment. The lesions of 84.5% of the patients were healed without complications, 3.9% had secondary lesions, and 11.6%, a lower proportion than in other studies, had functional limitations. Hereby, edema, category III ulcers, healing times >180 days, and limitation of movement at discharge constituted the main risk factors. Review of all BUD related documentation revealed a number of shortcomings, in particular concerning medical records. In view of these findings, standardization of procedures for creating of therapy plans, patient assessment and follow-up, as well as improved management of medical records are recommended to facilitate implementation of therapeutic measures to optimize treatment outcome and to allow for further evaluation.

## Introduction

Buruli ulcer disease (BUD), caused by *Mycobacterium ulcerans*, is a chronic, necrotizing skin disease which has been reported from more than 30 countries worldwide with a focus in West Africa [1]. BUD predominantly affects impoverished inhabitants of remote rural areas, approximately 50% of the cases are children <15 years [1–2]. Initially BUD manifests as painless nodule, plaque, papule, or edema followed by large, painless ulcerations with characteristically undermined edges [1–3]. Also cases with osteomyelitis occur [1–2, 4–5]. Lesions are divided into three categories (I: single lesions, <5 cm diameter; II: single lesions, 5–15 cm diameter; III: single lesions, >15 cm diameter, multiple lesions, lesions at critical sites, osteomyelitis) [2]. As a result of scarring and contractures emerging during the healing process, especially patients who are not treated early suffer long-term functional disability [1, 6]. As the mode of transmission of BUD has not been elucidated to date, proven strategies of prevention do not exist [1]. Early diagnosis and treatment are therefore core elements of BUD control which requires strong commitment of health workers at community level, laboratory confirmation of 70% of suspected cases by standardized diagnostic methods (preferably IS*2404* PCR), and standardized antimycobacterial treatment (rifampicin [R] in combination with streptomycin [S], alternatively clarithromycin [C] for eight weeks), if necessary complemented by surgery and/or physiotherapy [2, 7–9]. The WHO classified BUD as one of the currently five neglected tropical diseases (NTDs) in line for the “innovative and intensified disease management (IDM)” approach, demanding a major scaling up of active case detection, treatment, monitoring and surveillance [10].

Since the introduction of antimycobacterial combination therapy a number of clinical studies investigated the treatment outcome of BUD patients, in particular healing times, secondary lesions and functional limitations. Whereas several authors observed healing of lesions of more than 90% of patients receiving various antimycobacterial treatment regimens (RS8, RS4/RC4, RS2/RC6) within twelve months [11–13], information on the time to healing varies. Nienhuis et al. reported median healing times of category I lesions of 18 weeks, and 30 weeks for category II and III lesions respectively [12]. Sarfo et al. further specified median healing times for nodules of 8 weeks, for ulcers of overall 12 weeks (category I: 12 weeks; category II: 11 weeks; category III: 15.5 weeks), and edema ranging from 2–48 weeks [11], Phillips et al. described median healing times of 14 weeks (RS8) and 16 weeks (RS2RC6) [13], and Vincent et al. observed median healing times of 12.6 weeks [5]. Available data from various studies also suggest that healing of up to two thirds of patients occurs within about 25 weeks after onset of treatment [5, 12–14].

Whereas proven recurrences were non-existent [11–13] or below 2% [15], paradoxical reactions in terms of deterioration of lesions on antibiotic treatment or the appearance of secondary lesions during or after treatment, were described for individual patients [16–18] and for larger patient cohorts. Nienhuis et al. found an increase in lesion size in up to 80%, and secondary lesions in 6% of the patients participating in the BURULICO antimicrobial trial in Ghana [12, 19], O’Brien et al. described paradoxical reactions in 21% of an Australian patient cohort [20], and Phillips et al. reported 9% of paradoxical reactions in a Ghanaian patient cohort participating in a recent antimicrobial trial (RS2/RC6) [13]. Increases in lesion size were commonly observed during the first three months after onset of treatment [19–20], but also delayed paradoxical reactions in terms of new lesions occurring up to thirteen months after the end of antibiotic treatment are known [17–18].

Functional limitations were frequently observed. Data from two cohorts of laboratory confirmed BUD patients from Ghana treated between 2003 and 2005 (surgery with or without concomitant antibiotic treatment), and between 2004 and 2009 (antimycobacterial treatment with or without surgical intervention), suggested functional limitations in 27% and 33.3% of the patients [21–22]. A comparison of two patient cohorts from the Democratic Republic of the Congo treated between 2002 and 2004 (surgical treatment only) and 2005–2007 (the majority of patients underwent surgery, more than 50% also received antimycobacterial therapy) showed that 23.4% and 19.5% of the patients healed with complications [4]. A recent study from Benin analyzed a cohort of more than 1000 BUD patients treated between 2005 and 2011 with antimycobacterial combination therapy and surgery if required, and reported 22% permanent functional limitations one year after treatment [5].

Since the early 2000s, several investigators conducted in-depth assessments of functional limitations and identified important risk factors for their development, in particular location on joints and extremities of limbs, lesions >15 cm, and lesions at head and neck [21–28]. Beyond that, Vincent et al. recently established a specific profile of risk factors for BUD patients from Benin (edema, osteomyelitis, lesions >15 cm in diameter, multifocal lesions, healing times >107 days) and introduced the operational definition “severe Buruli ulcer” to earmark patients at risk for functional limitations for specific disability prevention measures [5].

In Togo, systematic BUD control was initiated in 2007. Whereas case finding, laboratory confirmation and antimycobacterial treatment have been fully implemented [29–30], accompanying POD (prevention of disability) measures as outlined by the WHO [6] are not yet sufficiently embedded in routine procedures, and treatment outcome has not been monitored.

This study presents the first analysis of treatment outcome of BUD patients in Togo, critically reviews procedures with a possible impact on the occurrence of complications, and provides recommendations for optimization of treatment success.

## Materials and Methods

### Ethical considerations

Ethical clearance was obtained through the national Togolese ethics committee (‘‘Comité de Bioéthique pour la Recherche en Santé”) at the University of Lomé (14/2010/CBRS) and the study was approved by the ‘‘Ministère de la Santé de la République Togolaise” Lomé, Togo (Ref. No. 0009/2011/MS/DGS/DPLET). Written informed consent (IC) was obtained in French, if necessary translated into local languages, from all study participants and/or their legal representatives if aged below 18 years.

### Management of BUD in Togo

In Togo, BUD control mainly operates through a network of district based CLTs (“Contrôleurs Lèpre-TB-Buruli”) and community based ASCs (“Agent Santé Communitaire”). CLTs regularly conduct sensitization activities in villages and schools, furthermore perform active case finding supported by ASCs who report patients with suspected BUD lesions to their corresponding CLT. Due to extended coverage of sensitization activities, self-referrals of patients to the nearest health post (“Unité de Soins Périphérique [USP]”) are on the rise. CLTs as well as USP head nurses (“[ICP] Infirmier Chef Poste”) refer clinically suspected BUD cases to the regional reference hospital (“Centre Hospitalier Régionale [CHR] Tsévié”). At CHR-Tsévié a specifically trained medical assistant (“point focal” [PF]) is in charge of further proceedings, such as physical examination, documentation on the WHO recommended BU 01.N and BU 01.R forms in case of suspected recurrences [31], sample collection and initiation of laboratory confirmation at the national hygiene institute (“Institute Nationale d’Hygiène [INH]”) according to standardized procedures [29–30]. Whereas most patients with category I (partially also category II) lesions are referred for outpatient treatment to USPs, the majority of patients with severe category II and category III lesions, and children <15 years in general are treated at CHR-Tsévié. Antimycobacterial treatment follows WHO recommendations and is complemented by surgical intervention if required [2]. Wound management at CHR-Tsévié is performed by nurses, at the USPs by the ICP, in both cases according to instructions of the PF. Patients who, according to the judgment of the PF, require physical therapy are referred to physiotherapists who provide treatment at the reference hospital, the USPs and also the patients homes. Currently 7 state examined physiotherapists are seconded to treatment of BUD patients.

### Study population

Eligible for the study were 199 PCR confirmed BUD patients originating from regions “Maritime”, “Savanes”, “Plateaux” and “Centrale” who were treated within the period from September 2007 to November 2013 with more than 6 months elapsed since the end of treatment. Inclusion and exclusion criteria are shown in Table 1.

**Table 1:** Inclusion and exclusion criteria.

Inclusion criteria	Exclusion criteria
IS*2404* PCR confirmed BUD patient of any age	Laboratory unconfirmed cases
Antimycobacterial treatment (RS8) was administered (with or without completion of 56 doses) with or without surgical interventions	Antimycobacterial treatment initiated before September 2007 or after November 2013
>6 months elapsed since the end of antimycobacterial treatment	<6 month elapsed since the end of antimycobacterial treatment
written informed consent provided	written informed consent not provided

Table 1 shows inclusion and exclusion criteria for study participants.

Clinical, epidemiological and treatment data were retrospectively compiled from existing databases of previous studies which contained information retrieved from standardized WHO BU 01.N and project specific laboratory data entry forms, and cross-checked with original paper forms [29–30].

### Study forms

To collect standardized data on treatment outcome a study specific form (S1 Form) was employed which consisted of several sections: A-D) clinical/epidemiological baseline and treatment data (taken from existing databases, prefilled prior to follow-up visits); E-F) information on location of suspected secondary lesions (recorded in the field) and clinical samples collected for laboratory diagnosis; G-H) assessment of limitations of movement (LOM) and impairment in daily activities (based on the questionnaire developed by Simonet V [32]; documented in the field); I) recommendations for further medical treatment (issued after data analysis). In addition, for patients with open wounds at follow-up the BU 01.R form and a clinical record form (as used routinely in Togo; S2 Form [30]) were filled in the field. S1 Table summarizes all parameters collected for analysis.

### Additional source materials

For patients with secondary lesions and functional limitations case histories were retrospectively retrieved from medical records, where available. The distance from the patients’ location of residence to CHR was obtained from logbooks of DAHW-T cars. In addition, documentation on physiotherapy was retrospectively reviewed, as far as accessible (“Fiche de bilan des patients atteints de l’ulcère de Buruli” and “Prévention des incapacités liées à l’UB—formulaire de base”; see S3 and S4 Forms).

### Follow-up visits

A total of 25 follow-up visits to 29 USPs (corresponding to the catchment area of 61 villages) were conducted in January-April 2013 (110 patients) and May-June 2014 (19 patients). In advance, patients were grouped according to location of residence and accessibility of the nearest USP and summoned by the responsible ICP upon instructions of the PF at a specific date. A field team (surgeon, physiotherapist, medical assistant and PF) enrolled study participants at the USPs and performed clinical examination and questioning according to the above described study form (S1 Form) if informed consent was provided. For patients unavailable to attend an attempt was made to retrieve them in their villages at a later date.

Patients whose lesions were healed without complications were discharged. From patients with open wounds at examination (in that context referred to as secondary lesions) lesions were measured and categorized according to WHO guidelines, and clinical samples were collected for microbiological analysis. Patients with anatomical impairment (including excessive scars and open wounds) were subjected to goniometric measurements according to the sagittal, frontal, and transverse rotation method (SFTR) [33] and scars were measured by the surgeon and medical assistant of the field team. Furthermore, these patients were questioned about the functional impairment in daily life according to the questionnaire in part H of the study form (S1 Form). Functional limitations were defined as BUD related anatomical impairment as determined by goniometry and/or measurement of scars and were classified in type I (i.e. anatomical impairment not hampering daily activities) and type II (i.e. anatomical impairment hampering daily activities).

### Microbiological analyses

Collection of swab samples, fine-needle aspirates or 3mm punch biopsies as well as microscopy and IS*2404* qPCR followed standardized procedures at the laboratories of the INH, Lomé accompanied by external quality assurance conducted at the Department for Infectious Diseases and Tropical Medicine, Munich, Germany, as recently described [30]. For regular bacteriological analysis, conducted in the accredited (COFRAC, ‘‘Comité Français d’Accréditation” according to NF EN ISO/CEI 17025 [version 2005]) bacteriology unit of the INH, swab samples were inoculated on Chapman (mannitol-salt [MSA]) agar, blood agar and nutrient broth (BioRad, Munich, Germany). Colonies indicative for *Staphylococcus aureus* were isolated from MSA agar, analyzed by Gram staining, catalase and coagulase test, and subjected to susceptibility testing using the Kirby-Bauer disc diffusion method (15 antimicrobials) on Müller-Hinton agar (BioRad) [34].

### Statistical analysis

The study design was a non-randomized clinical cohort study. Statistical analysis (chi-square test, including Fisher exact test) was carried out by EPIINFO 3.3.2. (CDC, Atlanta, GA, USA). The results of statistical analyses were presented by *P*-values. Significant differences were defined as *P*-values below 0.05.

## Results

### Patients enrolled and baseline data

Out of 199 BUD patients eligible for the study, 129 (64.8%) could be retrieved and enrolled as follow-up patients in the study. Among the 129 follow-up BUD patients, 46.5% were male. At the time of initial diagnosis 90 of 129 follow-up patients (69.8%) were below 15 years of age (range 2–68 years, median 10 years, interquartile range [IQ] 7–16 years). The patients originated from 6 districts of region “Maritime”. The distance from the place of residence to CHR-Tsévié was known for 120 patients (93.0%) and was 1–23 km for 47 patients (39.2%) and 24–135 km for 73 patients (60.8%). The duration of disease before clinical diagnosis was known for 128 patients (99.2%) and was 0–42 days for 81 patients (63.3%), and 43–3.600 days for 47 (36.7%) patients. Baseline data and details on statistical analyses are provided in S2 Table.

### Drop-out patients and baseline-data

Out of 199 BUD patients eligible for the study, 70 patients (35.2%) could not be enrolled (drop-outs). Forty-three patients (61.4%) had moved to an unknown address, 24 (34.3%) were not found and 3 were deceased (4.3%). Among the 70 drop-out patients, 52.9% were male (no significant difference with the follow-up patients). At the time of initial diagnosis 36 of 70 drop-out patients (51.4%) were above 15 years of age (range 2–65 years, median 15.5 years, IQ 8.3–28 years) and significantly older than the follow-up patients (*P*<0.01%). The drop-out patients originated from 6 districts of region “Maritime”, 2 districts of region “Plateaux”, 1 district of region “Centrale” and 1 district of region “Savanes”. The distance from the place of residence to CHR-Tsévié was known for 60 drop-out patients (85.7%) and was 1–23 km for 34 patients (56.7%) and 24–135 km for 26 patients (43.3%); the drop-out patients lived significantly closer to CHR Tsévié than the follow-up patients (*P* = 0.03%). The duration of disease before clinical diagnosis was known for 69 drop-out patients (98.6%) and was 0–42 days for 21 patients (30.4%) and 43–3.600 days for 48 (69.6%) patients; the drop-out patients had a significantly longer duration of disease than the follow-up patients (*P*<0.01). Baseline data and details on statistical analyses are provided in S2 Table.

### Characteristics of initial lesions

At the time of initial diagnosis 73 of the 129 follow-up patients (56.6%) had ulcers and 56 patients (43.4%) had non-ulcerative lesions (nodule, n = 19 [33.9%]; plaque, n = 26 [46.4%]; edema, n = 11 [19.6%], 10 edemas evolved into an ulcer). Fifty-nine patients (45.7%) had category I lesions, 44 patients had category II lesions (34.1%) and 26 patients (20.2%) had category III lesions. Four of the patients with category III lesions had multiple lesions [multiple ulcers, n = 2; ulcer and nodule, n = 1; ulcer and plaque, n = 1]). The localization of lesions was as follows: upper limbs, n = 51 (39.5%); lower limbs, n = 50 (39.8%); trunk/head, n = 28 (21.7%). Lesions of 45 patients (34.9%) involved joints (category I, n = 23 [51.1%]; category II, n = 18 [40.0%]; category III, n = 4 [8.9%]). LOM at time of initial diagnosis were not documented.

### Treatment and time to healing

Fourty-nine patients (38.0%; category I, n = 13; category II, n = 17; category III, n = 19) received antimycobacterial treatment at CHR-Tsévié, 35 of these patients (71.4%) underwent also surgery (excision and grafting, n = 11 [31.4%]; grafting, n = 23 [65.7%]; reconstructive surgery, n = 1 [2.9%]). Out of these 35 patients, 10 had category I lesions, 8 had category II lesions, and 17 had category III lesions. Eighty patients (62.0%) were referred to USPs (category I, n = 46; category II, n = 27; category III, n = 7) for antimycobacterial therapy.

Among the 129 follow-up BUD patients, 126 patients completed antibiotic therapy (97.7%), three patients (2.3%) did not (two patients were incompliant and for the third the reason was not known). LOM after the end of treatment were documented for 126 patients (97.7%). Out of them, 17 patients (13.5%; category I, n = 2; category II, n = 6; category III, n = 9) were discharged with LOM.

The time to healing was known for 124 patients (96.1%) and ranged from 1–146 days for 63 patients (50.8%; significant correlation with category I lesions [*P*<0.01]), and 147–784 days for 61 patients (49.2%; significant correlation with category III lesions [*P*<0.01]). Stratified into categories of lesions, 57 patients (46.0%) with category I lesions had a median healing time of 108 days (IQR: 93.5–149.5), 42 patients (33.9%) with category II lesions had a median healing time of 151 days (IQR: 125.8–208), and 25 patients (20.1%) with category III lesions had a median healing time of 256 days (IQR: 177–314). Among 41 patients with healing times of more than 180 days, we also observed a correlation with functional limitations (*P*<0.01).

### Physiotherapy

According to the BU 01.N forms, 117 out of the 129 follow-up BUD patients (90.7%) received physiotherapy; however for 76 of these patients (65.0%) detailed documentation and physiotherapeutic treatment protocols were not available. Nine follow-up BUD patients (7.0%) did not receive physiotherapy for unknown reasons and for three patients (2.3%) this information was not available. Eighteen patients (23.1%) were treated at CHR-Tsévié only, 60 patients (76.9%) at USPs/patients homes and 39 patients (33.3%) at both locations. The number of sessions was documented for 95 patients (81.2%): 24–99 sessions, n = 46 (48.4%); 100–520 sessions, n = 49 (51.6%). Detailed physiotherapeutical treatment protocols, however, did not exist.

### Findings at follow-up

Among the 129 follow-up BUD patients, the lesions of 109 patients (84.5%) were completely healed without any complications. Five patients (3.9%) had secondary lesions (2 of them in combination with functional limitations). Whereas *M*. *ulcerans* DNA was not detected in any of the lesions, strains of *S*. *aureus* were isolated from two patients (one of them revealed a methicillin resistant *S*. *aureus* [MRSA]), in four cases the etiology of secondary lesions remained unclear. Fifteen patients (11.6%) had functional limitations (type I, n = 5 [3.9%]; type II, n = 10 [7.8%]; two of them in combination with secondary lesions). From 80 patients (62.0%) scars were measured. Out of them, 22 patients (27.5%) had scars with a diameter of <5cm, 33 (41.3%) had scars with a diameter of 5–15cm, and 25 (31.3%) had scars with a diameter of >15cm.

### Risk factors for functional limitations

Among the clinical findings, functional limitations were significantly associated with healing times >180 days (*P*<0.01), edema (*P*<0.01), and category III lesions (ulcers >15cm or multiple lesions; *P*<0.01), and a documented LOM at time of discharge (*P*<0.01). Treatment related factors significantly associated with functional limitations were surgery (*P*<0.01) and hospitalization at CHR-Tsévié (*P*<0.01). S2 Table provides detailed risk factor analyses.

## Discussion

This study provides the first analysis of treatment outcome of BUD patients in Togo. The median times to healing as determined for various categories of patients lie within the range of values reported by other authors. Likewise, our data also suggest that the lesions of approximately two third of the patients healed within about 25 weeks as reported by other authors [5, 11–14]. The absence of proven recurrences in our study is also in line with the low or non-existing recurrence rate as observed by other investigators [11–13, 15]. As previously published, one patient of our study cohort had developed a delayed paradoxical reaction 10 months after the end of antimycobacterial treatment [18]. At the time of follow-up initial and secondary lesions were completely healed, the patient was therefore not included in the group of patients with complications. Five patients had secondary lesions at the time of clinical examination which may be related to delayed type paradoxical reactions—this is however purely speculative as the patients could not precisely indicate time of occurrence and clinical course of the lesions. From the lesions of two of these patients *S*. *aureus* strains, one of them MRSA, were isolated. Although this is the first reported case of MRSA from Togo, this finding was to be expected as investigators from the neighboring countries Ghana and Benin have recently shown that a high proportion of BUD lesions are colonized with *S*. *aureus*, and MRSA is frequently isolated [35–37]. The Togolese MRSA patient was treated with vancomycin and reportedly healed under antibiosis. It became however apparent that follow-up procedures for identification of such complications are lacking, furthermore, a concept for antibiotic management of super-infected BUD lesions does not exist.

A drawback of this study was that almost 35% of laboratory confirmed patients treated with standardized antimycobacterial treatment could not be retrieved at follow-up visits. According to medical records or BU 01.N forms, 60 of the drop-out patients (85.7%, out of them 58 patients without LOM [96.7%]) were completely healed at discharge. We could however not assess long-term sequelae among the drop-out cohort. To avoid these lost to follow-ups, which are likely to occur in mobile populations such as in Togo, we strongly recommend the introduction of standardized follow-up procedures for BUD patients in Togo.

Among the cohort of BUD patients retrieved for follow-up the percentage of functional limitations of 11.6% was lower than in other studies [4–5, 21–22]. However, we need to mention that, in the absence of formal definitions, we introduced an operational definition of type I and II functional limitations, therefore a direct comparison between our data and other studies may not be possible without restrictions. Our data suggest that edema and category III ulcers, a healing time >180 days as well as LOM at discharge constitute the main risk factors for functional limitations in Togolese BUD patients. The finding that hospitalization and surgical treatment at CHR Tsévié were also associated with functional limitations can be explained by the fact that 73% of patients with category III lesions were hospitalized at the reference center and 89% of them underwent surgery.

In analogy with the operational definition of a “severe Buruli ulcer” as established by Vincent et al. [5], we suggest to introduce criteria for the systematic identification of patients with increased risk for functional limitations also into clinical management of BUD in Togo. We propose to draw special attention to patients initially presenting with edema and category III ulcers, furthermore—although our data did not show a significant correlation—joint involvement as shown by other authors [21–23, 25–26].

A recent study emphasized the special importance of wound care for the prevention of BUD related functional limitations [38]. Although according to our data most Togolese patients with “severe Buruli ulcer” have already been hospitalized in CHR-Tsévié and received advanced wound management, we recommend making it a general rule. Optimal wound management should consist of daily cleansing with saline solution (in cases of severe exudation twice a day), removal of necrotic tissue, and use of vaseline dressing for prevention of drying of the wound. In addition, consistent implementation of the POD related essential health interventions as outlined by the WHO are required and necessitate intensified training programs for hospital staff, CLTs, ICPs and physiotherapists [6].

This study provided an excellent opportunity to review all BUD related documentation. Clinical, epidemiological and treatment records on BU 01.N forms were for the most part complete. The status of LOM at admission was however not documented, and information on evolution of wounds during treatment was not available. For that reason we were not able to retrospectively analyze the prevalence of early paradoxical reactions in terms of enlargement of wounds. Likewise, extensions of lesions were only known for the time of admission and it was impossible to keep track of lesions expanding over joints subsequent to initial diagnosis, which may explain the absence of a significant correlation between lesions over joints and functional limitations in our study cohort. Concerning surgery, operation reports were not available, and information on indication, type and frequency of surgical interventions was largely retrieved from handwritten notes and oral reports of PF and surgeons.

For more than 60% of the patients who allegedly had received physical therapy, written documentation was absent, and treatment protocols indicating the type of exercises performed did not exist. Therefore, conclusions on the impact of physical therapy on prevention and clinical improvement of functional limitations could not be drawn in this study.

In view of these findings, optimization of procedures accompanying or following antimycobacterial treatment are highly recommended. Improvement of documentation of surgical and physiotherapeutic interventions is required and shall be facilitated through filing maps.

Furthermore, to standardize concomitant physiotherapeutic measures, at the time of admission each patient should be seen by a physical therapist to decide on the general requirement of physical therapy and to prepare a treatment schedule, if applicable. Upon completion of antimycobacterial treatment, the PF at CHR-Tsévié and specially trained CLTs at the USPs respectively, should conduct a standardized assessment for each patient to decide on discharge and/or further therapeutic measures. The individual package of measures for each patient shall be defined in a treatment schedule which is regularly monitored by PF and CLTs. As a general rule, all patients should be followed until complete healing of the wound, afterwards at least once per year for a five year period, thus facilitating timely recognition of two further risk factors for functional limitations, i.e. prolonged healing times and LOM at/after discharge, as well as delayed paradoxical reactions. Regular feedback on fulfillment of treatment measures and results of follow-up visits to the PNLUB-LP (“Programme National de Lutte contre l’Ulcère de Buruli, la Lèpre et le Pian”) is considered mandatory to enhance the transparency of the system and to allow for further evaluation and improvement.

## Supporting Information

S1 Table List of parameters collected for analysis. (DOCX)

S2 Table Data of the study cohort comprising 199 PCR confirmed BUD patients. (DOCX)

S1 Form Clinical follow-up form. (PDF)

S2 Form BUD clinical record and laboratory data entry form. (PDF)

S3 Form “Fiche de bilan des patients atteints de l’ulcère de Buruli”. (PDF)

S4 Form ”Prévention des incapacités liées à l’UB—formulaire de base”. (PDF)
