# Supplementary material for: Treatment Outcome of Patients with Buruli Ulcer Disease in Togo
Source: PLoS Negl Trop Dis. 2015 Oct 16;9(10):e0004170. doi: 10.1371/journal.pntd.0004170 (PMC4608783; doi:10.1371/journal.pntd.0004170)
Supplement: S1 Table — (DOCX) [file pntd.0004170.s001.docx]

**Table S1.** List of parameters collected for analysis

| **Source** | **Independent variables** | **Dependent variables** |
| --- | --- | --- |
| BU 01.N form, data retrieved from medical records; S1 Form, part A | General information | Name of patient |
|  |  | Adress (village/town) |
|  |  | District |
|  |  | Region |
|  |  | Country |
|  |  | Age at diagnosis (years) |
|  |  | Sex |
|  |  | Profession |
|  |  | Patient classification at diagnosis (new case/recurrence) |
|  |  | Date of clinical diagnosis or admission |
|  |  | Date of complete healing |
|  |  | Duration of illness before seeking care |
| BU 01.N form, data retrieved from medical records | Clinical history at diagnosis | Limitation of movement at any joint |
| BU 01.N form, data retrieved from medical records; S1 Form, part B | Type of lesion(s) | non-ulcerative lesions: |
|  |  | Nodule |
|  |  | Oedema |
|  |  | Osteomyelitis |
|  |  | Plaque |
|  |  | Papule |
|  |  | Ulcerative lesion |
| BU 01.N form, data retrieved from medical records; S1 Form, part B | Category of lesion(s) | Category I |
|  |  | Category II |
|  |  | Category III, multiple lesions, lesions at critical sites, osteomyelitis |
| BU 01.N form, data retrieved from medical records | Location of lesion(s) | Head and neck |
|  |  | Upper limb |
|  |  | Lower limb |
|  |  | Thorax |
|  |  | Back |
|  |  | Abdomen |
|  |  | Buttocks and perineum |
|  |  | Critical sites (eye, breast, genitalia) |
| BU 01.N form, data retrieved from medical records; S1 Form, part C | Type of treatment | Dressings |
|  |  | Antibiotics |
|  |  | Surgery |
|  |  | POD (prevention of disability) |
| BU 01.N form, data retrieved from medical records | Dosage(s) | Rifampicin (mg) |
|  |  | Streptomycin (g) |
|  |  | Other (name; mg) |
|  |  |  |
| BU 01.N form, data retrieved from medical records; S1 Form, part G | Treatment outcome | 1a: antibiotic treatment completed |
|  |  | 1b: antibiotic treatment not completed |
|  |  | 2a: healed without surgery |
|  |  | 2b: healed with surgery |
|  |  | 3a: healed without limitation of movement at any joint |
|  |  | 3b: healed with limitation of movement at any joint |
|  |  | 4: referred for further treatment |
|  |  | 5: lost of follow up (died) |
| S1 Form,  part A and D | Clinical findings at follow-up | BCG scar (yes/no) |
|  |  | Buruli ulcer occurrence in family (yes/no) |
|  |  | Suspected recurrence |
|  |  | Multiple lesions |
|  |  | Occurrence of a lesion in the period between discharge and follow-up (trauma, burn or other) |
|  |  | Duration of new illness/lesion |
|  |  | Location of lesion(s) (upper limbs, lower limb, other) |
| S1 Form, part E; BU 01.R form and S2 Form | Suspected secondary lesion | Clinical form (nodule, oedema, osteomyelitis, plaque, papule, ulcer) |
|  |  | Recurrence |
|  |  | Location of lesion  Size of lesion  Category of lesion |
|  |  | Photo documentation of lesion |
| S1 Form, part F | Collection of clinical samples for laboratory confirmation | Swab sample |
|  |  | Fine needle aspirate |
|  |  | 3mm punch biopsy |
| S1 Form, part G | Functional limitation | Functional limitation before initial treatment |
|  |  | Location of functional limitation before initial treatment |
|  |  | Spontaneous development without interventions |
|  |  | Development caused by an intervention |
|  | Cause of functional limitation | Multiple interventions |
|  |  | Interventions due to BUD |
|  | Extent of functional limitation | Level of deficit of movement |
|  |  | Goniometric measurement of range of movement |
|  |  | The patient is handicapped |
|  |  | and deprived from performing new activities |
|  |  | measurement of scar (__ cm diameter) |
| S1 Form, part H | Subjective functional impairment | **the patient has difficulties to:** |
|  |  | wash him/herself and take care of their appearance |
|  |  | dress him/herself |
|  |  | use toilet facilities |
|  |  | **the patients has difficulties:** |
|  |  | walking short distances |
|  |  | walking longer distances |
|  |  | walking on uneven ground or slopes |
|  |  | using a vehicle |
|  |  | running |
|  |  |  |
|  |  | **the patient has difficulties:** |
|  |  | running chores (grocery shopping) |
|  |  | prepare meals |
|  |  | taking care of children |
|  |  | taking care of the household |
|  |  | **the patient has difficulties :** |
|  |  | during school activities |
|  |  | during business/work activities |
|  |  | during free time activities |
|  |  | during social/community based activities |
| Table S1 shows all parameters collected for analysis. | | |
