# Supplementary material for: Treatment Outcome of Patients with Buruli Ulcer Disease in Togo
Source: PLoS Negl Trop Dis. 2015 Oct 16;9(10):e0004170. doi: 10.1371/journal.pntd.0004170 (PMC4608783; doi:10.1371/journal.pntd.0004170)
Supplement: S2 Table — (DOCX) [file pntd.0004170.s002.docx]

**Table S2. Data of the study cohort comprising 199 PCR confirmed BUD patients**

| **Variables** | | **Study cohort (%)** | **Follow up BUD patients** | | | ***P*-value^a^** | **Drop out BUD patients (%)** | ***P*-value^b^** |
| --- | --- | --- | --- | --- | --- | --- | --- | --- |
|  |  |  | **All (%)** | **With functional limitation (%)** | **Without functional limitation (%)** |  |  |  |
| 1. Sample size | BUD patients | 199 (100) | 129 (100) | 15 (100) | 114 (100) | N.A. | 70 (100) | N.A. |
|  | Proportion (%) | (100) | (64.82) | (7.54) | (57.29) | N.A. | (35.18) | N.A. |
| 2. Sex | Female | 102 (51.26) | 69 (53.49) | 8 (53.33) | 61 (53.51) | 0.99 | 33 (47.14) | 0.39 |
|  | Male | 97 (48.74) | 60 (46.51) | 7 (46.67) | 53 (46.49) |  | 37 (52.86) |  |
| 3. Age at time of clinical diagnosis, in years | Range (minimum-maximum) | 2-68 | 2-68 | 6-50 | 2-68 | N.A. | 2-65 | N.A. |
|  | Age group: 2-11 | 98 (49.25) | 74 (57.36) | 10 (66.67) | 64 (56.14) | 0.44 | 24 (34.29) | <0.01* |
|  | Age group: 12-68 | 101 (50.75) | 55 (42.64) | 5 (33.33) | 50 (43.86) |  | 46 (65.71) |  |
|  | Median (interquartile range) | 12 (7-21) | 10 (7-16) | 9 (7-14) | 11 (7-16.75) | N.A. | 15.5 (8.25-28) | N.A. |
| 4. Location of residence | Région Maritime | 195 (97.99) | 129 (100) | 15 (100) | 114 (100) | N.A. | 66 (94.29) | <0.01* |
|  | District Afagnan | 1 (0.50) | 0 | 0 | 0 | N.A. | 1 (1.43) | 0.35^c^ |
|  | District Ave | 1 (0.50) | 1 (0.78) | 0 | 1 (0.88) | 1.00^c^ | 0 | 1.00^c^ |
|  | District Bas Mono | 1 (0.50) | 0 | 0 | 0 | N.A. | 1 (1.43) | 0.35^c^ |
|  | District Golfé | 4 (2.01) | 3 (2.33) | 1 (6.67) | 2 (1.75) | 0.31^c^ | 1 (1.43) | 1.00^c^ |
|  | District Lacs | 2 (1.01) | 2 (1.55) | 1 (6.67) | 1 (0.88) | 0.22^c^ | 0 | 0.54^c^ |
|  | District Vo | 6 (3.02) | 4 (3.10) | 1 (6.67) | 3 (2.63) | 0.39^c^ | 2 (2.86) | 1.00^c^ |
|  | District Yoto | 98 (49.25) | 70 (54.26) | 7 (46.67) | 63 (55.26) | 0.53 | 28 (40.00) | 0.06 |
|  | District Zio | 82 (41.21) | 49 (37.98) | 5 (33.33) | 44 (38.60) | 0.69 | 33 (47.14) | 0.21 |
|  | Région Plateaux | 2 (1.01) | 0 | 0 | 0 | N.A. | 2 (2.86) | 0.12^c^ |
|  | District Ani | 1 (0.50) | 0 | 0 | 0 | N.A. | 1 (1.43) | 0.35^c^ |
|  | District Ogou | 1 (0.50) | 0 | 0 | 0 | N.A. | 1 (1.43) | 0.35^c^ |
|  | Région Centrale | 1 (0.50) | 0 | 0 | 0 | N.A. | 1 (1.43) | 0.35^c^ |
|  | District Sotouba | 1 (0.50) | 0 | 0 | 0 | N.A. | 1 (1.43) | 0.35^c^ |
|  | Région Savanes | 1 (0.50) | 0 | 0 | 0 | N.A. | 1 (1.43) | 0.35^c^ |
|  | District Dapaong | 1 (0.50) | 0 | 0 | 0 | N.A. | 1 (1.43) | 0.35^c^ |
| 5. Distance from location of residence to CHR, in km | Not known | 19 (9.55) | 9 (6.98) | 0 | 9 (7.89) | 0.60^c^ | 10 (14.29) | 0.09 |
|  | Known | 180 (90.45) | 120 (93.02) | 15 (100) | 105 (92.11) |  | 60 (85.71) |  |
|  | Circuit of 1-135 | 180 (100) | 120 (100) | 15 (100) | 105 (100) | N.A. | 60 (100) | N.A. |
|  | Circuit of 1-23 | 81 (45.00) | 47 (39.17) | 5 (33.33) | 42 (40.00) | 0.62 | 34 (56.67) | 0.03* |
|  | Circuit of 24-135 | 99 (55.00) | 73 (60.83) | 10 (66.67) | 63 (60.00) |  | 26 (43.33) |  |
|  | Median (interquartile range) | 24 (21-27) | 24 (21-27) | 27 (21-39) | 24 (21-27) | N.A. | 21.75 (18-27) | N.A. |
|  | Mean  (standard deviation) | 26.66  (15.28) | 27.47  (15.83) | 36.99  (32.22) | 26.11  (11.49) | N.A. | 25.03  (14.12) | N.A. |
|  | 95% confidence interval | 24.43; 28.88 | 24.65; 30.29 | 20.69; 53.30 | 23.91; 28.31 | N.A. | 21.48; 28.57 | N.A. |
| 6. Duration of disease before clinical diagnosis, in days | Not known | 2 (1.01) | 1 (0.78) | 0 | 1 (0.88) | 1.00^c^ | 1 (1.43) | 1.00^c^ |
|  | Known | 197 (98.99) | 128 (99.22) | 15 (100) | 113 (99.12) |  | 69 (98.57) |  |
|  | 0-3,600 | 197 (100) | 128 (100) | 15 (100) | 113 (100) | N.A. | 69 (100) | N.A. |
|  | 0-42 | 102 (51.78) | 81 (63.28) | 7 (46.67) | 74 (65.49) | 0.16 | 21 (30.43) | <0.01* |
|  | 43-3,600 | 95 (48.22) | 47 (36.72) | 8 (53.33) | 39 (34.51) |  | 48 (69.57) |  |
|  | Median (interquartile range) | 42 (21-112) | 28 (21-60) | 56 (21-98) | 28 (21-60) | N.A. | 90 (30-168) | N.A. |
|  | Age group: 2-11 years | 97 (100) | 74 (100) | 10 (100) | 64 (100) | N.A. | 23 (100) | N.A. |
|  | 0-42 | 67 (69.07) | 57 (77.03) | 6 (60.00) | 51 (79.69) | 0.22^c^ | 10 (43.48) | <0.01* |
|  | 43-3,600 | 30 (30.93) | 17 (22.97) | 4 (40.00) | 13 (20.31) |  | 13 (56.52) |  |
|  | Age gr.: 12-68 years | 100 (100) | 54 (100) | 5 (100) | 49 (100) | N.A. | 46 (100) | N.A. |
|  | 0-42 | 35 (35.00) | 24 (44.44) | 1 (20.00) | 23 (46.94) | 0.37^c^ | 11 (23.91) | 0.03* |
|  | 43-3,600 | 65 (65.00) | 30 (55.56) | 4 (80.00) | 26 (53.06) |  | 35 (76.09) |  |
| 7. Number of lesions | Single lesion | 195 (97.99) | 125 (96.90) | 14 (93.33) | 111 (97.37) | 0.39^c^ | 70 (100) | 0.30^c^ |
|  | Multiple lesions | 4 (2.01) | 4 (3.10) | 1 (6.67) | 3 (2.63) |  | 0 |  |
| 8. Clinical presentation | Ulcerative lesions | 118 (59.30) | 73 (56.59) | 10 (66.67) | 63 (55.26) | 0.40 | 45 (64.29) | 0.29 |
|  | Non-ulcerative lesions | 81 (40.70) | 56 (43.41) | 5 (33.33) | 51 (44.74) |  | 25 (35.71) |  |
|  | Non-ulcerative lesions | 81 (100) | 56 (100) | 5 (100) | 51 (100) | N.A. | 25 (100) | N.A. |
|  | Plaque | 34 (41.98) | 26 (46.43) | 0 | 26 (50.98) | 0.055^c^ | 8 (32.00) | 0.12 |
|  | Nodule | 29 (35.80) | 19 (33.93) | 1 (20.00) | 18 (35.29) | 0.65^c^ | 10 (40.00) | 0.93 |
|  | Edema | 17 (20.99) | 11 (19.64) | 4 (80.00) | 7 (13.73) | <0.01^c^* | 6 (24.00) | 0.99 |
|  | Papule | 1 (1.23) | 0 | 0 | 0 | N.A. | 1 (4.00) | 0.35^c^ |
|  | No sec. ulceration^d^ | 186 (93.47) | 119 (92.25) | 11 (73.33) | 108 (94.74) | 0.02^c^* | 67 (95.71) | 0.55^c^ |
|  | Secondary ulceration ^d^ | 13 (6.53) | 10 (7.75) | 4 (26.67) | 6 (5.26) |  | 3 (4.29) |  |
|  | Age group: 2-11 years | 98 (100) | 74 (100) | 10 (100) | 64 (100) | N.A. | 24 (100) | N.A. |
|  | No sec. ulceration^d^ | 89 (90.82) | 65 (87.84) | 6 (60.00) | 59 (92.19) | 0.02^c^* | 24 (100) | 0.11^c^ |
|  | Secondary ulceration^d^ | 9 (9.18) | 9 (12.16) | 4 (40.00) | 5 (7.81) |  | 0 |  |
|  | Age gr.: 12-68 years | 101 (100) | 55 (100) | 5 (100) | 50 (100) | N.A. | 46 (100) | N.A. |
|  | No sec. ulceration^d^ | 97 (96.04) | 54 (98.18) | 5 (100) | 49 (98.00) | 1.00^c^ | 43 (93.48) | 0.33^c^ |
|  | Secondary ulceration^d^ | 4 (3.96) | 1 (1.82) | 0 | 1 (2.00) |  | 3 (6.52) |  |
| 9. Localization of lesions | Upper limbs | 87 (43.72) | 51 (39.53) | 6 (40.00) | 45 (39.47) | 0.97 | 36 (51.43) | 0.11 |
|  | Lower limbs | 74 (37.19) | 50 (38.76) | 8 (53.33) | 42 (36.84) | 0.22 | 24 (34.29) | 0.53 |
|  | Trunk/head | 38 (19.10) | 28 (21.71) | 1 (6.67) | 27 (23.68) | 0.19^c^ | 10 (14.29) | 0.20 |
| 10. Category of lesions | Category I | 85 (42.71) | 59 (45.74) | 3 (20.00) | 56 (49.12) | 0.03* | 26 (37.14) | 0.24 |
|  | Category II | 71 (35.68) | 44 (34.11) | 3 (20.00) | 41 (35.96) | 0.22 | 27 (38.57) | 0.53 |
|  | Category III | 43 (21.61) | 26 (20.16) | 9 (60.00) | 17 (14.91) | <0.01* | 17 (24.29) | 0.50 |
|  | Age group: 2-11 years | 98 (100) | 74 (100) | 10 (100) | 64 (100) | N.A. | 24 (100) | N.A. |
|  | Category I | 46 (46.94) | 37 (50.00) | 1 (10.00) | 36 (56.25) | <0.01* | 9 (37.50) | 0.29 |
|  | Category II | 31 (31.63) | 21 (28.38) | 2 (20.00) | 19 (29.69) | 0.71^c^ | 10 (41.67) | 0.23 |
|  | Category III | 21 (21.43) | 16 (21.62) | 7 (70.00) | 9 (14.06) | <0.01^c^ | 5 (20.83) | 0.94 |
|  | Age gr.: 12-68 years | 101 (100) | 55 (100) | 5 (100) | 50 (100) | N.A. | 46 (100) | N.A. |
|  | Category I | 39 (38.61) | 22 (40.00) | 2 (40.00) | 20 (40.00) | 1.00^c^ | 17 (36.96) | 0.91 |
|  | Category II | 40 (39.60) | 23 (41.82) | 1 (20.00) | 22 (44.00) | 0.39^c^ | 17 (36.96) | 0.62 |
|  | Category III | 22 (21.78) | 10 (18.18) | 2 (40.00) | 8 (16.00) | 0.22^c^ | 12 (26.09) | 0.34 |
| 11. Joint involvement | Not occurred | 135 (67.84) | 84 (65.12) | 9 (60.00) | 75 (65.79) | 0.66 | 51 (72.86) | 0.27 |
|  | Occurred | 64 (32.16) | 45 (34.88) | 6 (40.00) | 39 (34.21) |  | 19 (27.14) |  |
|  |  |  |  | 1 (9.09) | 0 |  | 2 (10.53) |  |
| 12. Location of treatment | CHR and USP | 1 (0.50) | 1 (0.78) | 0 | 1 (0.88) | 1.00^c^* | 0 | 1.00^c^* |
|  | CHR or USP | 198 (99.50) | 128 (99.22) | 15 (100) | 113 (99.12) |  | 70 (100) |  |
|  | CHR or USP | 198 (100) | 128 (100) | 15 (100) | 113 (100) | N.A. | 70 (100) | N.A. |
|  | CHR only (with hospitalization) | 86 (43.43) | 49 (38.28) | 13 (86.67) | 36 (31.86) | <0.01* | 37 (52.86) | 0.048* |
|  | USP only (without hospitalization) | 112 (56.57) | 79 (61.72) | 2 (13.33) | 77 (68.14) |  | 33 (47.14) |  |
| 13. Antibiotic treatment | Not known | 1 (0.50) | 0 | 0 | 0 | N.A. | 1 (1.43) | 0.35^c^ |
|  | Known | 198 (99.50) | 129 (100) | 15 (100) | 114 (100) |  | 69 (98.57) |  |
|  | Completed or not | 198 (100) | 129 (100) | 15 (100) | 114 (100) | N.A. | 69 (100) | N.A. |
|  | Completed | 193 (97.47) | 126 (97.67) | 15 (100) | 111 (97.37) | 1.00^c^ | 67 (97.10) | 1.00^c^ |
|  | Not completed | 5 (2.53) | 3 (2.33) | 0 | 3 (2.63) |  | 2 (2.90) |  |
| 14. Surgery | Not conducted | 145 (72.86) | 94 (72.87) | 4 (26.67) | 90 (78.95) | <0.01^c^* | 51 (72.86) | 1.00 |
|  | Conducted | 54 (27.14) | 35 (27.13) | 11 (73.33) | 24 (21.05) |  | 19 (27.14) |  |
|  | Conducted | 54 (100) | 35 (100) | 11 (100) | 24 (100) | N.A. | 19 (100) | N.A. |
|  | Grafting | 51 (94.44) | 34 (97.14) | 10 (90.91) | 24 (100) | 0.31^c^ | 17 (89.47) | 0.29^c^ |
|  | No grafting | 3 (5.56) | 1 (2.86) | 1 (9.09) | 0 |  | 2 (10.53) |  |
| 15. Location of physiotherapy | No physiotherapy | 24 (12.06) | 12 (9.30) | 0 | 12 (10.53) | 0.36^c^ | 12 (17.14) | 0.16 |
|  | Physiotherapy | 175 (87.94) | 117 (90.70) | 15 (100) | 102 (89.47) |  | 58 (82.86) |  |
|  | Physiotherapy | 175 (100) | 117 (100) | 15 (100) | 102 (100) | N.A. | 58 (100) | N.A. |
|  | Both: CHR and USP | 58 (33.14) | 39 (33.33) | 10 (66.67) | 29 (28.43) | 0.01* | 19 (32.76) | 0.92 |
|  | One: CHR or USP/homes | 117 (66.86) | 78 (66.67) | 5 (33.33) | 73 (71.57) |  | 39 (67.24) |  |
|  | One: CHR or USP/homes | 117 (100) | 78 (100) | 5 (100) | 73 (100) | N.A. | 39 (100) | N.A. |
|  | CHR only | 36 (18.09) | 18 (23.08) | 3 (60.00) | 15 (20.55) | 0.08^c^ | 18 (46.15) | 0.02* |
|  | USP/homes only | 81 (40.70) | 60 (76.92) | 2 (40.00) | 58 (79.45) |  | 21 (53.85) |  |
| 16. Physiotherapy: number of sessions | Not known | 53 (26.63) | 34 (26.36) | 1 (6.67) | 33 (28.95) | 0.11^c^ | 19 (27.14) | 0.96 |
|  | Known | 146 (73.37) | 95 (73.64) | 14 (93.33) | 81 (71.05) |  | 51 (72.86) |  |
|  | 24-520 sessions | 146 (100) | 95 (100) | 14 (100) | 81 (100) | N.A. | 51 (100) | N.A. |
|  | 24-99 sessions | 71 (48.63) | 46 (48.42) | 4 (28.57) | 42 (51.85) | 0.11 | 25 (49.02) | 0.95 |
|  | 100-520 sessions | 75 (51.37) | 49 (51.58) | 10 (71.43) | 39 (48.15) |  | 26 (50.98) |  |
|  | Median (interquartile range) | 100 (65-140) | 100 (65-160) | 180 (80-237.5) | 90 (65-140) | N.A. | 100 (70-128) | N.A. |
| 17. Limitation of movement (LOM) at discharge | Not known | 7 (3.52) | 3 (2.33) | 1 (6.67) | 2 (1.75) | 0.31^c^ | 4 (5.71) | 0.22 |
|  | Known | 192 (96.48) | 126 (97.67) | 14 (93.33) | 112 (98.25) |  | 66 (94.29) |  |
|  | Status of LOM at discharge known | 192 (100) | 126 (100) | 14 (100) | 112 (100) | N.A. | 66 (100) | N.A. |
|  | No LOM at discharge | 167 (86.98) | 109 (84.50) | 5 (35.71) | 104 (92.86) | <0.01* | 58 (87.88) | 0.97 |
|  | LOM at discharge | 25 (13.02) | 17 (13.18) | 9 (64.29) | 8 (7.14) |  | 8 (12.12) |  |
|  | Age group: 2-11 years | 98 (100) | 74 (100) | 10 (100) | 64 (100) | N.A. | 24 (100) | N.A. |
|  | No LOM at discharge | 83 (84.69) | 63 (85.14) | 2 (20.00) | 61 (95.31) | <0.01^c^* | 20 (83.33) | 1.00^c^ |
|  | LOM at discharge | 15 (15.31) | 11 (14.86) | 8 (80.00) | 3 (4.69) |  | 4 (16.67) |  |
|  | Age gr.: 12-68 years | 101 (100) | 55 (100) | 5 (100) | 50 (100) | N.A. | 46 (100) | N.A. |
|  | No LOM at discharge | 91 (90.10) | 49 (89.09) | 4 (80.00) | 45 (90.00) | 0.45^c^ | 42 (91.30) | 0.75^c^ |
|  | LOM at discharge | 10 (9.90) | 6 (10.91) | 1 (20.00) | 5 (10.00) |  | 4 (8.70) |  |
| 18. Time to complete healing, in days | Not known | 15 (7.54) | 5 (3.88) | 3 (20.00) | 2 (1.75) | 0.02^c^* | 10 (14.29) | 0.03* |
|  | Known | 184 (92.46) | 124 (96.12) | 12 (80.00) | 112 (98.25) |  | 60 (85.71) |  |
|  | 1-146 | 92 (50.00) | 63 (50.81) | 3 (25.00) | 60 (53.57) | 0.06 | 29 (48.33) | 0.75 |
|  | 147-784 | 92 (50.00) | 61 (49.19) | 9 (75.00) | 52 (46.43) |  | 31 (51.67) |  |
|  | Median (interquartile range) | 146.5 (106-211.50) | 143.5 (103.75-210) | 311 (225.50-355.75) | 134 (102.50-191.50) | N.A. | 148.5 (114.75-216.75) | N.A. |
|  | Category I:  Time to heal known | 79 | 57 | 2 | 55 | N.A. | 22 | N.A. |
|  | Category I: Median (interquartile range) | 114 (95-150) | 108 (93.50-149.50) | 324 (30-618) | 108 (93-149) | N.A. | 126 (102.00-170.50) | N.A. |
|  | Category II:  Time to heal known | 66 | 42 | 2 | 40 | N.A. | 24 | N.A. |
|  | Category II: Median (interquartile range) | 151 (126.50-209.50) | 151 (125.75-208) | 121.5 (100-143) | 152.5 (125.75-209.50) | N.A. | 151 (128.50-209.25) | N.A. |
|  | Category III:  Time to heal known | 39 | 25 | 8 | 17 | N.A. | 14 | N.A. |
|  | Category III: Median (interquartile range) | 240 (161.50-311) | 256 (177-314) | 314.5 (305.75-355.75) | 187 (158-259) | N.A. | 192 (134.50-269.50) | N.A. |
| 19. Time difference between date of patient healed and date of follow-up, in days | Not known or N.A. | N.A. | 10 (7.75) | 5 (33.33) | 5 (4.39) | N.A. | N.A. | N.A. |
|  | Known | N.A. | 119 (92.25) | 10 (66.67) | 109 (95.61) |  | N.A. |  |
|  | 24-1,930 | N.A. | 119 (100) | 10 (100) | 109 (100) | N.A. | N.A. | N.A. |
|  | 24-576 | N.A. | 60 (50.42) | 5 (50.00) | 55 (50.46) | 0.98 | N.A. | N.A. |
|  | 577-1,930 | N.A. | 59 (49.58) | 5 (50.00) | 54 (49.54) |  | N.A. |  |
|  | Mean  (standard deviation) | N.A. | 708.50  (473.97) | 637.80  (588.98) | 714.99  (464.80) | N.A. | N.A. | N.A. |
|  | 95% confidence interval | N.A. | 623.34; 793.66 | 272.75; 1,002.85 | 627.73; 802.25 | N.A. | N.A. | N.A. |
| 20. Death during study period | No death | 196 (98.49) | 129 (100) | 15 (100) | 114 (100) | 1.00^c^ | 67 (95.71) | 0.04^c^* |
|  | Death | 3 (1.51) | 0 | 0 | 0 |  | 3 (4.29) |  |

Table S2 shows the data of the study cohort comprising 199 PCR confirmed BUD patients.

N.A., not applicable. CHR, Centre Hospitalier Régional (in Tsévié, Togo). USP, ”Unité de Soins Périphérique” (peripheral health posts, in different locations in Togo).

^a^ *P*-values: Comparing the follow-up BUD patients with functional limitation and the follow-up BUD patients without functional limitation.

^b^ *P*-values: Comparing all follow-up BUD patients (with and without functional limitation) and the drop-out BUD patients.

^c^ Fisher exact chi-square test.

^d^ Secondary ulceration of initial edema under antimycobacterial treatment.

*Significant differences were defined as *P*-values <0.05.
