# Supplementary material for: Treatment Outcome of Patients with Buruli Ulcer Disease in Togo
Source: PLoS Negl Trop Dis. 2015 Oct 16;9(10):e0004170. doi: 10.1371/journal.pntd.0004170 (PMC4608783; doi:10.1371/journal.pntd.0004170)
Supplement: S1 Form — (PDF) [file pntd.0004170.s003.pdf]

H: EVALUATION DES LIMITATIONS D'ACTIVITES (V.Simonet)

Cotation :	2	oui, la personne éprouve des difficultés dans cette tâche
	1	non, la personne n'éprouve pas de difficultés dans cette tâche
	0	La personne n'effectue habituellement pas cette tâche

TACHES		
1)	La personne éprouve-t-elle des difficultés à s'occuper seule des soins la concernant :	
	Description	Cotation
	Pour se laver, soigner son apparence	
	Pour s'habiller	
	Pour utiliser les latrines	
2)	La personne éprouve-t-elle des difficultés à se déplacer :	
	Pour marcher sur de courtes distances à se déplacer seule	
	Pour marcher sur de longues distances	
	Pour marcher sur des surfaces inégales ou en pente	
	Pour se déplacer avec un véhicule	
	Pour courir	
3)	La personne éprouve-t-elle des difficultés dans sa vie domestique	
	Pour faire le marché	
	Pour préparer les repas	
	Pour s'occuper des enfants	
	Pour entretenir le foyer	
4)	La personne épreuve-t-elle des difficultés dans sa vie sociale, professionnelle ou scolaire	
	Pour réaliser ses activités scolaires	
	Pour réaliser ses activités lucratives habituelles	
	Pour participer aux activités de loisir	
	Pour participés aux activités communautaires	

I: REEDUCATION	Quelles mesures devraient être réalisées ?		
	RIEN A FAIRE	PHYSIOTHERAPIE	AUTRES INTERVENTION
**Déficit de Mobilité**	0 ☐	1 ☐	2 ☐
**Cicatrice**	0 ☐	1 ☐	2 ☐
**Amputation**	0 ☐	1 ☐	2 ☐

Date : 2013 Signature

BuruliVac- Pays: Togo - Suivi des Malades

Formulaire du Suivi **Date de visite :** ....2013 **USP** ....

A: ID du patient .... **Age actuel** ....**ans**

Nom .... **Prénom** ....

Village .... **District** ....

Sexe : M ☐ F ☐	Cicatrice BCG : Oui ☐ Non ☐	UB dans la famille : Oui ☐ Non ☐
Consentement : Oui ☐ Non ☐		Formulaires OMS UB01-R : Oui ☐ Non ☐

B:Buruli précédent - Présentation clinique

☐ Nodule	☐ Papule	☐ Plaque	☐ Lésion unique
☐ Ulcéré	☐ Œdème	☐ Ostéomyélite	☐ Lésions multiples ( )

Catégorie : ☐ I ☐II ☐III

C: Traitement

**1er Traitement**	RF/STR : ☐	Autre : ☐	Chirurgie : ☐
	Date début du traitement :		Date Fin de traitement :
**2eme Traitement**	RF/STR : ☐	Autre : ☐	Chirurgie : ☐
	Date début du traitement :		Date Fin de traitement :
Sorti gueri:☐	Sorti non gueri:☐	Quelles sequelles?	

D: Résultat

**Suspect Récidive/Rechute :**	☐ Récidive ☐ Rechute
**Suspect Incapacité :**	☐ Non ☐ Oui ➝ Document Incapacité
**Durée de la nouvelle maladie :**	.... semaines
**Lésion douteuse :** Oui ☐ Non ☐	**Consultation** recommandée dans 4 semaines : Oui ☐ Non ☐
**Lésion intermédiaire :** Oui ☐	**Laquelle ?** ☐Traumatisme (1) ☐Brûlure (2) ☐Autre (3)
**Lésion multiples :**	☐ Membre inf. (1) ☐ Membre sup. (2) ☐ autre Localité (3)

S'IL N'YA AUCUNE PATHOLOGIE LIBERER LA PERSONNE EVALUEE

E : Localisation et lésion (Suspect Récidive/Rechute)

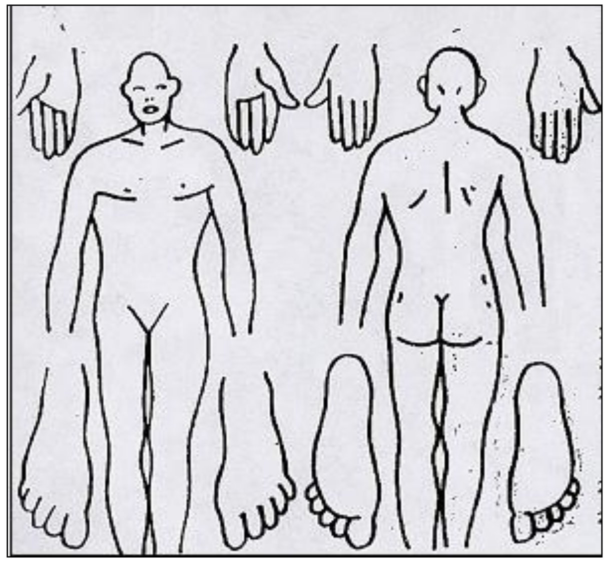

Œdème	Plaque	Nodule	Ulcére
(OE) = 1	(P) = 2	(N) = 3	(U) = 4

☐	Récidive : Même endroit comme précédent

Localisation		
Membre inf. (a)	Membre sup. (b)	Autres (c)

Documentation photographique	
☐ OUI	☐ NON
**Date :**	
**Signature :**	

F: Echantillons cliniques suspects rechute- MIC[1] PCR[2] RNA[3] **WP6 :** □oui □ non

A) Nodule, Plaque, Œdème:	B) Ulcère:	C)Autres + ulcère chronique (borde cicatrisée)
1. FNA (tube 300ml CLS) pour PCR<br>2. FNA (frotti pour ZN-Microscopie)<br>3. FNA (tube PANTA 500µl)pour PCR (DNA+RNA)	1. Swab(tube 700ml CLS) pour PCR<br>2. Swab(frotti pour ZN-Microscopie)<br>3. Swab(tube PANTA 500µl) pour PCR (DNA+RNA)	1. FNA (tube 300ml CLS) pour PCR<br>2. FNA (frotti pour ZN-Microscopie)<br>3. FNA (tube PANTA 500µl) pour PCR (DNA+RNA)

A Nodule, Plaque, Œdème

☐

Ø du nodule/plaque ..../...............mm

WP6 (*PCR **MIC *** RNA)			
	AAF1* ☐	Biopsie punch* ☐	Biopsie chirurgicale * ☐
	AAF2**☐		
	AAF3***☐		

B Ulcère

☐ Ø de l'ulcère ..../.......... mm Catégorie....................................

WP6 (*PCR ** MIC ***RNA)			
Ecouvillon1* ☐	AAF1*☐	Biopsie punch* ☐	Biopsie chirurgicale * ☐
Ecouvillon2** ☐	AAF2**☐		Biopsie chirurgicale** ☐
Ecouvillon3*** ☐	AAF3*** ☐		

C Autres (Ostéomyélite) + ulcère chronique (bord cicatrisé)

☐

WP6 (*PCR **MIC)		
Ecouvillon 1 ☐		Biopsie chirurgicale * ☐
Ecouvillon 2 ☐		Biopsie chirurgicale** ☐

UB vérifié : □oui □ non

DATES ET HISTOIRE DU MALADE	Date de la visite 2013	ID :
Nom :	Prénom :	
USP :	Village :	District :
Buruli précédent :		

Œdème (1) □ Plaques (2) □ Nodule (3) □ Ulcére (4) □ CategorieI II III

G: INCAPACITÉ (INCAP) :

INCAP. antérieur au traitement précédent/Tsévié :	Oui (1)□	Non (0)□
Si oui laquelle ?		
Localisation :	Membre inf. (1) □	Membre sup. (2) □ / Autres Localisations (3) □
Développement spontanée sans intervention au cours du traitement :	Oui (1)□	Non (0)□
Développement lié à l'intervention/Tsévié :	Oui (1)□	Non (0)□
Si oui lié à laquelle ?		
Excision (1) □	Greffe (2) □	Autres (3) □
Multiples interventions :	Oui (1)□	Non (0)□
Interventions liées à l'UB :	Oui (1)□	Non (0)□
Documentation Photo :	Oui (1)□	Non (0)□

DEFICIT DE MOBILITE

DOCUMENTATION DE L'INCAPACITE : Indiquer comme suivant:

Cicatrice ‡‡‡ Déficit de Mobilité →→ Amputation—— Œdème O

Au quel niveau ?		
1)		
2)		
Mesurer le déficit articulaire à l'aide du **Goniomètre**		
1)		
2)		
Le déficit de mobilité est resté :		
□	Tolérable (1)	
□	Augmenté (2)	
La personne est génée :	Oui (1) □	Non (0) □
Elle a repris de nouvelles Activités :	Oui (1) □	Non (0) □
Les mesures de la cicatrice (cm x cm):	Longueur :	Largeur :

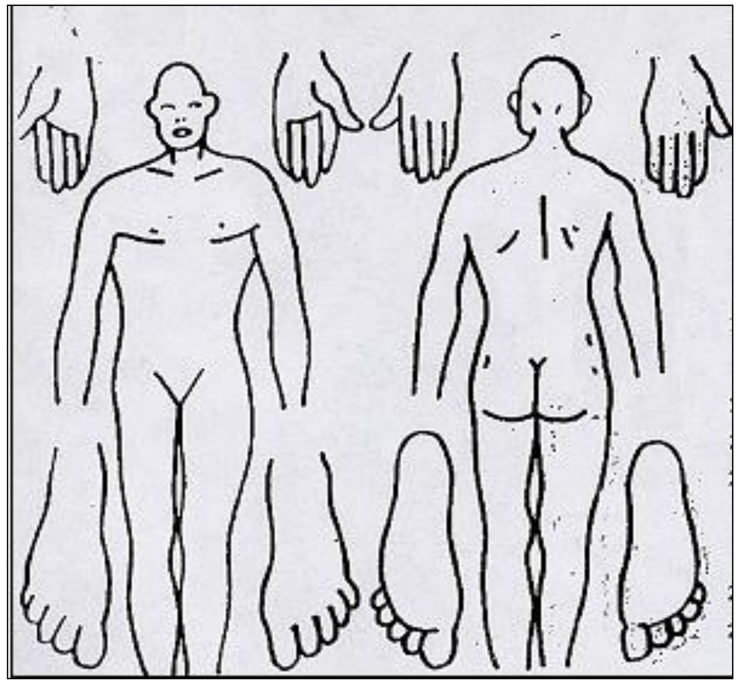

Si le déficit de mobilité de la personne a augmenté et/ou si elle est beaucoup génée et limitée dans ses activités quotidiennes continuer en détails par : « L'Evaluation des Limitations d'Activité » d'après V. Simonet (tourner svp)

Date : 2013 Signature
