# Supplementary material for: Treatment Outcome of Patients with Buruli Ulcer Disease in Togo
Source: PLoS Negl Trop Dis. 2015 Oct 16;9(10):e0004170. doi: 10.1371/journal.pntd.0004170 (PMC4608783; doi:10.1371/journal.pntd.0004170)
Supplement: S3 Form — (PDF) [file pntd.0004170.s005.pdf]

T/KE/194/10/N/DT

FICHE DE BILAN KINE DES PATIENTS ATTEINTS DE L'ULCERE DE BURULI

Numéro du dossier : .... Bilan effectué le : 12/05/10 par : [redacted]

I. DONNEES PERSONNELLES

1. Nom et prénom (s) : [redacted]

2. Sexe : Masculin ☒ Féminin ☐

2. Age :
- De 0 à 15 ans ☒
- De 16 à 20 ans ☐
- De 21 à 45 ans ☐
- De 46 à 60 ans ☐
- 60 ans et + ☐

4. Adulte situation matrimoniale
- Marié ☐
- Célibataire ☐
- Divorcé (e) ☐
- Veuf (ve) ☐

NB : Enfant = moins de 15 ans

5. Adulte situation professionnelle
- A une activité ☐
- Est sans activité ☐

Enfant scolarité
- Est scolarisé ☐
- N'est pas scolarisé ☒

6. Provenance : Région : Ma[illegible] Préfecture : YOTO Ville ou village : TCHEKPO ANAGALI

7. Traitement antérieur (Cocher)			
Centre de santé	X	Autre	
Médecine traditionnelle		Aucun	

II. BILAN/EVALUATION

1. Atteinte des fonctions et structures organiques

Atteinte	Cocher			
1.1 Œdème		1.1.1 Mesure :		
		1.1.2 Qualité (Cocher):	1.1.2.1 (Dur	
			1.1.2.2 Mou	
1.2 Plaie	X	1.2.1Couleur : Jaunâtre		
		1.2.2 Taille : (cocher)	1.2.2.1 Moins de 5cm	X
			1.2.2.2 De 5 à 15 cm	
			1.2.2.3 Plus de 15 cm	
1.3 Cicatrice		1.3.1 Couleur :		
		1.3.2 Mobilité :		
		1.3.3 Densité :		
		1.3.4 Taille :		
		1.3.5 Adhérence : (cocher)	1.3.5.1 X (pas adhérent)	
			1.3.5.2 XX (moyennement adhérent)	
			1.3.5.3 XXX (Adhérent)	
1.4 Plaque				
1.5 Nodule				
1.6 Déficit de mobilité				
1.7 Amputation				
1.8 Déformation				

Œdème	(symbole)
Plaque	P
Nodule	N
Plaie	(symbole)
Cicatrice	xxx
Déficit de mobilité	(symbole)
Amputation/ déformation	—

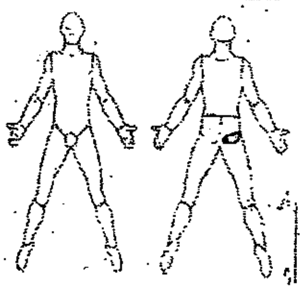

2. Bilan de la douleur à la mobilisation			
2.1 Lieu :	Fesse droite.		
2.2 Moment :	position couchée sur le dos		
2.3 Intensité (cocher) :	2.3.1 De 0 à 3 (faible)	X	
	2.3.2 De 4 à 7 (modérée)		
	2.3.3 De 8 à 10 (forte)		

Autres remarques sur les problèmes de sensibilité :

3. Localisation de l'atteinte (Cocher)			
3.1 Tête		3.8 Tronc et tête	
3.2 Tronc		3.9 Tronc et membre inférieur	
3.3 Membre inférieur		3.10 Tronc et membre supérieur	
3.4 Membre supérieur		3.11 Tête, tronc, et membre inférieur	
3.5 Membre inférieur et supérieur		3.12 Tête, tronc, et membre supérieur	
3.6 Membre supérieur et tronc		3.13 Tête, membre inférieur, et membre supérieur	
3.7 Membre inférieur et tronc		3.14Tronc, membre inférieur, et membre supérieur	
