# Supplementary material for: Treatment Outcome of Patients with Buruli Ulcer Disease in Togo
Source: PLoS Negl Trop Dis. 2015 Oct 16;9(10):e0004170. doi: 10.1371/journal.pntd.0004170 (PMC4608783; doi:10.1371/journal.pntd.0004170)
Supplement: S4 Form — (PDF) [file pntd.0004170.s006.pdf]

T/KG/096/08/N/DT
N° 4
Libéré

# Prévention des Incapacités liées à l'UB
## Formulaire de base

**Données sur le bénéficiare :**
Nom : [redacted]
Age : 14 ans
Village : Gape Aboozlokopé
Aire de santé : Womegba
District : Zio
Province :
Contact téléphonique :

Sexe : ☒ (M) ☐ (F)

☒ Ecolier/étudiant
☐ Au foyer
☐ Activité professionnelle
☐ Sans activité

☐ Marié(e)
☐ Divorcé(e)/séparé(e)
☐ Célibataire
☐ Veuf /veuve
☐ A des enfants

Date présumée du début de l'affection : Janvier 2007

Niveau de scolarité : CM II (monte en 6e)
Remarques sur la situation professionnelle/sociale/familiale :

**Traitement antérieur :**
☐ centre de santé
☒ médecine traditionnelle
☐ autre :
☐ aucun

**Interventions médicales**
☒ Antibiothérapie : Date début : 22/08/08 Date fin :
☒ Pansements. Date fermeture de la plaie :
☐ Excision, date :
☐ Greffe : date :
☐ Amputation : date, lieu :
☐ Chirurgie reconstructive : intervention, date et lieu :
☐ Autre :

**Interventions de rééducation et réadaptation**
☐ Enseignement à la personne et à sa famille des interventions nécessaires pour se prendre en charge soi-même
☐ Lutte contre l'œdème
☐ Prise en charge des cicatrices
☒ Exercices pour le gain de mobilité
☐ Confection d'attelles
☐ Pose de prothèse : date, lieu :
☐ Moyen auxiliaire pour usage à long terme : type et date de l'obtention :

☒ Hospitalisation : Date entrée : 20/08/08 Date sortie : 20/11/08
☐ Suivi ambulatoire : Date début : Date fin :

**Evaluation initiale :** Date : 26/08/08 Score de mobilité fonctionnelle : 15

| | |
|---|---|
| Œdème | ⌐ |
| Plaque | P |
| Nodule | N |
| Plaie | O |
| Cicatrice | xxx |
| Déficit de mobilité | ↗ |
| Amputation/déformation | — |

Limitations d'activité :
0 = 0
1 = 8
– = 7

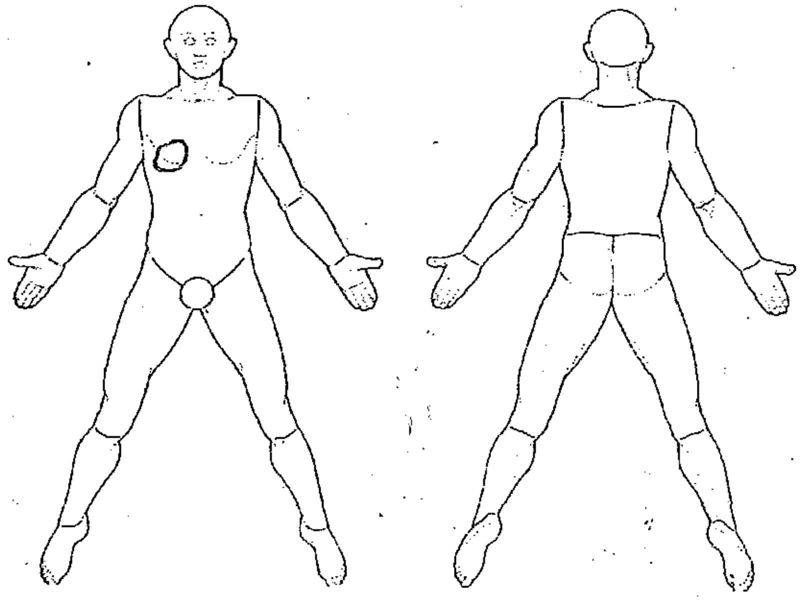

**Evaluation finale :** Date : Score de mobilité fonctionnelle :

Nom du référent pour le centre de référence : [redacted]
Nom du référent pour le centre de santé périphérique :
Nom du délégué de santé :
